# Treatment of endometrial cancer from 2000 to 2020 in Germany: a retrospective population based cohort study

**DOI:** 10.1007/s00432-024-05772-9

**Published:** 2024-05-27

**Authors:** Thomas Papathemelis, Olaf Ortmann, Cynthia Kohl, Petra Neuser, Kees Kleihues-van Tol, Monika Klinkhammer-Schalke, Peter Ugocsai, Christina Barbara Walter, Miriam Rottmann, Catherine Real, Christina Justenhoven, Gabriele Robers, Constanze Schneider, Michael Gerken, Andrea Sackmann, Soo-Zin Kim-Wanner

**Affiliations:** 1 Department of Gynecology and Obstetrics, Hospital St. Marien Amberg, Amberg, Germany; 2 Department of Gynecology and Obstetrics, University Medical Centre Regensburg, Regensburg, Germany; 3 Hessian Cancer Registry, Hessian Office for Health and Care, Frankfurt, Germany; 4 Arbeitsgemeinschaft Deutscher Tumorzentren E.V. (ADT), 14057 Berlin, Germany; 5 Tumor Center Regensburg, Institute of Quality Management and Health Services Research of the University of Regensburg, Regensburg, Germany; 6 Department of Women’s Health, University Women’s Hospital Tübingen, Tubingen, Germany; 7 Bavarian Cancer Registry, Regional Centre Munich, Bavarian Health and Food Safety Authority (LGL), Munich, Germany; 8 Cancer Registry North Rhine-Westphalia, Bochum, Germany; 9 Cancer Registry of Rhineland-Palatinate in the Institute for Digital Health Data, Mainz, Germany; 10 Cancer Registry Mecklenburg-Western Pomerania, Greifswald, Germany; 11 Cancer Registry Brandenburg-Berlin, Cottbus, Germany

**Keywords:** Overall survival, Outcome, Multimodal therapy, Surgery, Radiotherapy, Chemotherapy, Retrospective cohort study

## Abstract

**Purpose:**

Endometrial cancer (EC) is one of the most common malignancies among women in western countries. This study aimed to assess data on patient treatment in Germany throughout two decades to evaluate the development and effect of surgery, radiation, and chemotherapy.

**Methods:**

This retrospective registry study included 34,349 EC patients diagnosed between 2000 and 2020. Patients were classified into five risk groups. Overall survival was analyzed by Kaplan–Meier method as well as univariable and multivariable Cox regression to evaluate risk factors and treatment options.

**Results:**

Over the study period, minimal invasive surgery was used more often compared to open surgery and was associated with better overall survival. Patients with advanced EC were more likely to receive multimodal therapy.

Patients with intermediate risk EC had a good prognosis upon surgery, which further improved when radiotherapy was added. High-risk patients showed poorer prognosis but clearly benefited from additional radiotherapy. Survival of elderly high-risk patients with a non-endometrioid histology was improved when chemotherapy was added to surgery and radiotherapy.

**Conclusion:**

Our study includes a large analysis of data from German clinical cancer registries on the care of endometrial cancer during two decades. We observed an increase of minimal invasive surgery. There is evidence that minimal invasive surgery is not inferior to open surgery. Adjuvant radio- and chemotherapy further improves survival depending on risk group and age.

**Supplementary Information:**

The online version contains supplementary material available at 10.1007/s00432-024-05772-9.

## Introduction

With an incidence of more than 400,000 cases per year, endometrial cancer (EC) is one of the most common malignancies affecting female reproductive organs in western countries (Crosbie et al. 2022; Sung et al. 2021; World Health Organization. 2020). In 2019, Germany registered 10,860 new cases. The vast majority of these EC patients were diagnosed at a median age of 67 years (Zentrum für Krebsregisterdaten, Robert Koch-Institut 2024). Risk factors for EC include obesity, hypertension, hyperestrogenism and diabetes mellitus (Arnold et al. 2015; Colombo et al. 2016; Raglan et al. 2019). Staging of EC is performed according to the International Federation of Gynecology and Obstetrics (FIGO) classification (Edey and Murdoch 2010; Pecorelli 2009). The tumor stage at diagnosis has major impact on the five-year survival rates. For FIGO stages I and II, the prognosis is very good with a survival rate of more than 95% (Bock et al. 2018; Yen et al. 2020), whereas stage III and IV show poor prognosis with substantially reduced survival rates to 68% and 17%, respectively (Colombo et al. 2016). The German Centre for Cancer Registry Data reported a relative overall five-year survival rate for EC patients of 78% in Germany (Robert Koch-Institut 2024).

European and German treatment guidelines on EC recommend surgery for early-stage low risk tumors, and adjuvant chemotherapy and radiation for high-risk or advanced stage tumors (Colombo et al. 2016; Concin et al. 2021; Emons and Steiner 2018). The optimal treatment strategy is unclear. The guidelines recommend different systemic treatments for recurrent disease. However, the prognosis of advanced or recurrent EC is poor.

This study aimed to investigate routine data on treatment of EC patients over the last two decades, analyze the development of EC treatment and evaluate the compliance to EC guidelines. Furthermore, we intended to add evidence on risk group dependent effects of different therapeutic approaches.

## Materials and methods

### Study design

The study was designed as a retrospective cohort study and evaluated pseudonymized patient data from various German cancer registries covering the period from January 1, 2000, to December 31, 2020. The complete dataset contains 34,349 individuals from all parts of Germany, representing over 25% of all EC cases in the country (Schultz et al. 2023). The obtained data provides information on patients’ demographics (age, year of death, last follow-up), diagnosis (year, histology, TNM classification) and oncological care (type and duration of treatment) according to the German requirements of clinical cancer registration (Tol 2023).

### Study cohort

**Fig. 1:**
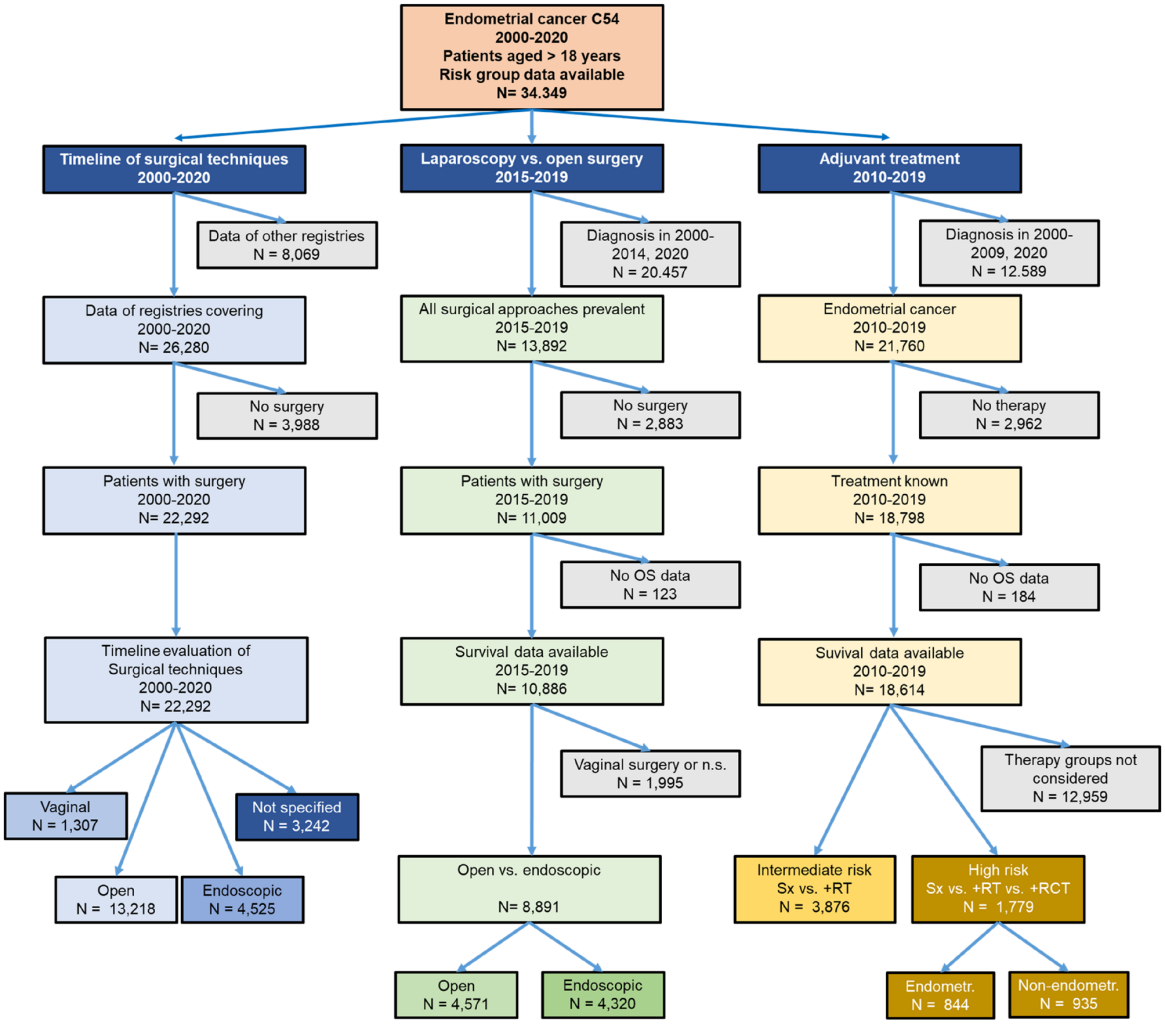
Flowchart of patient selection criteria for evaluation of surgical approaches and adjuvant therapy in this study

Patients from 23 cancer registries from 12 federal countries in Germany were included. Selection criteria for the study cohort were a minimum age of 18 years at diagnosis, and residency in the federal state of the reporting cancer registry. Further inclusion criteria were diagnosis of EC (ICD10: C54), localization of ICD-O-3 = 540, 541, 542, 543, 548 or 549 and histology behavior code of 3, excluding sarcomas, soft tissue tumors, mesothelioma, Kaposi's sarcoma. Patients with multiple tumors were excluded. In addition, patients with limited information for risk group stratification were excluded. In total, 34,349 patients were eligible for further analysis (s. Fig. 1). For trend analysis, data from registries that did not cover the complete study period from 2000 to 2020 and required information on treatment modalities were not included. For analyses considering risk group stratification the investigated cohort were restricted to years of diagnosis from 2010 to 2019 covering the revised FIGO classification from 2009, which shows an impact on the distribution of the risk groups (s. Supplementary Fig. 1) (Abu-Rustum et al. 2011; Pecorelli 2009). For survival analyses data with limited treatment information were excluded. For the comparison of surgery techniques (minimally invasive vs. open) data were restricted to the period after implementation of endoscopic surgery to years of diagnosis from 2015 to 2019. A detailed description of the study cohort is shown in Fig. 1.

### Selection of risk groups

All patients were classified based on the ESGO/ESTRO/ESP guidelines into five risk groups, which are summarized in Supplementary Table 1 (Concin et al. 2021). Molecular classification was not available and therefore not considered in the risk group classification. The ‘low risk’ group comprised patients with diagnosed endometrioid EC of stage FIGO IA, with grading G1 or G2 and negative LVSI. The ‘intermediate risk’ group included patients who were diagnosed with negative LVSI and (i) endometrioid EC of stage FIGO IB with grading G1 or G2, (ii) endometrioid EC of stage FIGO IA and grading G3, or (iii) non-endometrioid EC of stage FIGO IA with histology including serous, clear cell, undifferentiated or mixed cell carcinoma, or carcinosarcoma. The ‘high-intermediate’ risk group included patients with (i) endometrioid EC of stage FIGO I and LVSI, regardless of grading and myometrial invasion (ii) endometrioid EC of stage FIGO IB and grading G3, or (iii) non-endometrioid EC of stage FIGO II. The ‘high-risk’ group comprised patients with (i) non-endometrioid EC of stage FIGO I-IVA with myometrial invasion and histology including serous, clear cell, undifferentiated or mixed cell carcinoma, or carcinosarcoma, or (ii) non-endometrioid EC of stage FIGO III/IVA. The ‘advanced metastatic’ group contained patients who were diagnosed with (i) endometrial cancer of stage FIGO III/IVA and residual disease, or (ii) endometrial cancer with distant metastases of stage FIGO IVB.

### Statistical analysis

Descriptive statistical analyses were performed. Continuous data are described as means, median, minimum, and maximum values, while categorical data were presented as frequency counts and percentages. Overall survival (OS) time was calculated from the date of cancer diagnosis to the date of death regardless of cause in months or last date alive. Patients with limited follow-up data (< one month) were not included. Right censoring was performed 60 months from the date of diagnosis and at a maximum cut-off date of December 31st, 2019. OS was analyzed by Kaplan–Meier method, univariable, and multivariable Cox regression to compare primary treatment modalities (surgery (SX) vs. surgery and radiotherapy (RT) vs. chemotherapy (CTX) or radiochemotherapy (RCT)) in different risk groups. Hazard ratios (HR) for OS was considered significant if the confidence interval (CI) excluded 1, and the *p* value of the log-rank test was < 0.05.

Risk-adjustment was performed in multivariable analyses to adjust for confounding factors: age at diagnosis (< 70 years, ≥ 70 years), risk group (low, intermediate, high-intermediate, high, advanced metastatic), lymph node involvement (N status), LVSI (L status), vascular invasion (V status), residual disease (R status), grading, therapy (SX, SX + RT, SX + CTX, SX + RCT). All statistical analyses and graphical visualizations were performed using STATA 17 (StataCorp. 2021. Stata Statistical Software: Release 17. College Station, TX: StataCorp LLC).

## Results

### Type of surgery

**Fig. 2:**
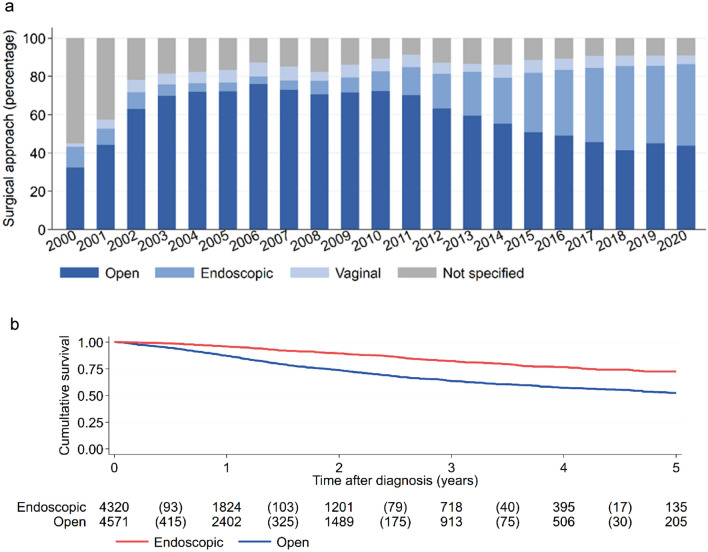
Surgical approach impact on overall survival. **a** Number of EC patients who were subjected to different surgical approaches per year of diagnosis. **b** Impact of surgical approach on overall survival over five years after diagnosis

22,292 patients included in the study were subjected to different surgical procedures (s. Fig. 2a). Of note is the relative change of case numbers for the individual surgical approaches over the study period. Based on the available data, open surgery was the most common approach in 2006 (75.9%), but hence started to decline to slightly above 40% in 2018 and beyond. By contrast, the fraction of minimal invasive surgery increased from 4.06% in 2006 to 44.0% in 2018, indicating a clear shift from open to minimal invasive surgery. The proportion of vaginal surgery remained with fractions from 1.7% (2000) to 7.3% (2006) overall relatively constant throughout the study period. The fraction of unspecified surgical approaches was 18.5% in 2003 and decreased to 9.3% in 2017 and remained on this level ever since.

### Development and effects of surgical approaches on overall survival

The dataset for survival analysis following surgery included 10,886 EC patients in five different risk groups who underwent various surgical approaches between 2015 and 2019. Most patients in the ‘low risk’ group underwent endoscopic surgery (53.4%), whereas only 27.0% underwent open surgery. In all other risk groups, patients predominantly underwent open surgery. This fraction of patients increased over the risk groups up to 68.7% in the ‘advanced metastatic’ risk group. By contrast, the fraction of patients who underwent endoscopic surgery declined to a minimum of 11.0% in the patient group associated with the highest risk, i.e. ‘advanced metastatic’. Table 1 shows the characteristics of 8,891 patients who underwent open or endoscopic surgery, excluding 1,995 patients subjected to vaginal or unspecified interventions.

**Table 1:** Effect of surgical treatment (open vs. endoscopic) of EC patients according to risk factors between 2015 and 2019

	Total	Surgical approach		HR	95% CI		p value
		Open (%)	Endoscopic (%)				
Age							
< 70 years	4924	2395 (49)	2529 (51)	1.000			
70 + years	3967	2176 (55)	1791 (45)	2.437	2.167	2.740	< 0.001
N status							
N0	6988	3476 (50)	3512 (50)	1.000			
N1	839	710 (85)	129 (15)	1.091	0.934	1.274	0.272
Not known	1064	385 (36)	679 (64)	1.729	1.453	2.057	< 0.001
V status							
V0	7478	3715 (50)	3763 (50)	1.000			
V1	630	489 (78)	141 (22)	1.313	1.124	1.534	0.001
Not known	783	367 (47)	416 (53)	1.361	0.852	2.173	0.198
L status							
L0	6495	3019 (46)	3476 (54)	1.000			
L1	1630	1194 (73)	436 (27)	1.230	1.060	1.427	0.006
Not known	766	358 (47)	408 (53)	0.957	0.589	1.556	0.860
Grading							
G1	3585	1305 (36)	2280 (64)	1.000			
G2	2793	1290 (46)	1503 (54)	1.227	1.033	1.457	0.020
G3	2322	1826 (79)	496 (21)	1.565	1.294	1.893	< 0.001
Not known	191	150 (79)	41 (21)	2.071	1.551	2.764	< 0.001
Histology							
Endometrioid	7459	3406 (46)	4053 (54)	1.000			
Non-endometrioid	1432	1165 (81)	267 (19)	1.160	1.002	1.342	0.046
Risk group							
Low	3679	1235 (34)	2444 (66)	1.000			
Intermediate	2088	1108 (53)	980 (47)	1.314	0.009	1.070	1.614
High-intermediate	1266	723 (57)	543 (43)	1.505	0.000	1.196	1.894
High	1285	1011 (79)	274 (21)	2.402	0.000	1.908	3.023
Advanced met	573	494 (86)	79 (14)	5.089	0.000	3.998	6.478
Total	8891	4571 (51)	4320 (49)	0.725	0.634	0.828	< 0.001

To evaluate the outcome of the surgical approach on overall survival, the analysis focused on patients who underwent open or endoscopic surgery, excluding the low numbers of patients who were subjected to other types of surgical approaches (s. Table 1).

Univariable survival analysis showed a clear statistical difference between open and endoscopic surgery, indicating that endoscopic intervention is associated with an improved overall survival (HR = 0.420; 95% CI = 0.372–0.475; p < 0.001) (s. Fig. 2b). The 5-year-survival rates were 73% and 50% for endoscopic and open surgery, respectively (s. Supplementary Table 2).

Multivariable statistical analysis supported this observation (Cox; HR = 0.725; 95% CI = 0.634–0.828; p < 0.001) (Table 1). In addition, the analysis revealed statistically significant differences for patient age (p < 0.001 for age of 70 years and older), V status (p = 0.001), L status (p = 0.006), grading (G2, p = 0.020; otherwise, p < 0.001), histology (p = 0.046), and risk group (p = 0.009 for intermediate; otherwise, p < 0.001).

### Effect of adjuvant radiotherapy in intermediate risk EC

The cohort dataset included 3,876 EC patients who were classified as intermediate risk. The majority underwent surgery only (n = 2082; 53.7%), whereas the other subset received radiotherapy in addition (n = 1794; 46.3%). Patients were followed up after surgery for an estimated median of 29.73 months. Table 2 shows the patient characteristics.

**Table 2:** Effect of therapy and risk factors on overall survival in EC patients with intermediate risk

	Total	SX + RT (%)	SX (%)	HR	95% CI		p value
Age							
< 70 years	1700	893 (52)	807 (48)	1.000			
70 + years	2176	901 (41)	1275 (59)	3.015	2.518	3.609	< 0.001
V status							
V0	3401	1587 (47)	1814 (53)	1.000			
V1	77	29 (38)	48 (62)	1.866	1.272	2.738	0.001
Not known	398	178 (45)	220 (55)	0.823	0.357	1.899	0.648
L status							
L0	3418	1591 (47)	1827 (53)	1.000			
L1	54	23 (43)	31 (57)	1.274	0.791	2.054	0.319
Not known	404	180 (46)	224 (55)	1.01	0.441	2.315	0.981
Grading							
G1	1315	571 (43)	744 (57)	1.000			
G2	1439	752 (52)	687 (48)	1.226	1.02	1.473	0.030
G3	1046	447 (43)	599 (57)	1.395	1.107	1.759	0.005
Not known	76	24 (32)	52 (68)	1.137	0.715	1.809	0.587
Histology							
Endometrioid	3270	1582 (48)	1688 (52)	1.000			
Non-endometrioid	606	212 (35)	394 (65)	1.399	1.097	1.785	0.007
Total	3876	1794 (46)	2082 (54)	2.211	1.889	2.587	< 0.001

*SX* surgery, *RT* radiotherapy, *HR* hazard ratio, *95% CI* 95% confidence interval, *V status* vascular status, *L status* lymphovascular status

**Fig. 3:**
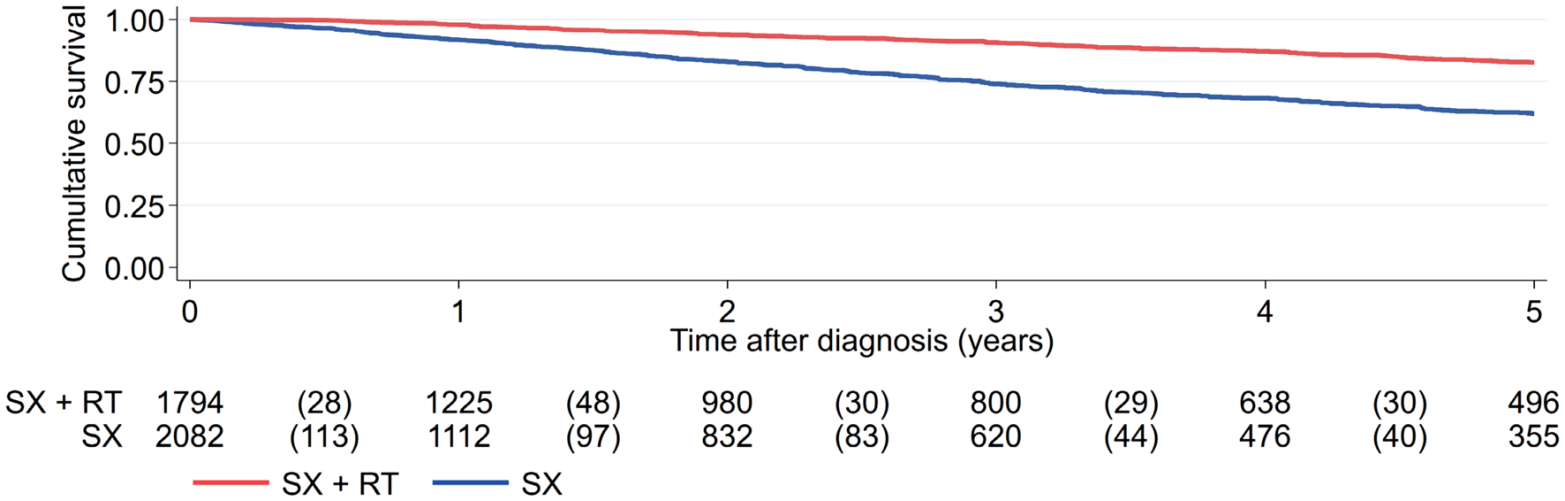
Overall survival for intermediate risk patients who received surgery with or without radiotherapy (*SX* surgery, *RT* radiotherapy)

Univariable survival analysis showed a clear statistical difference between both treatment groups, indicating that surgery alone shows a worse overall survival compared to adding radiotherapy to surgery (HR for surgery alone vs surgery + RT = 2.387; 95% CI = 2.044–2.787; p < 0.001) (s. Fig. 3). The addition of radiotherapy resulted in an improved 5-year OS of 20% with 62.0% for surgery alone compared to 82.6% for adjuvant radiotherapy.

Multivariable statistical analysis supported this observation (HR for surgery alone vs surgery + RT = 2.211; 95% CI = 1.889–2.587; p < 0.001) (Table 2) regardless of the histological subgroup (data not shown). In addition, the analysis revealed statistically significant differences for patient age years (p < 0.001), V status (p < 0.001), grading (G2, p < 0.001; G3, p < 0.001), histology (p = 0.007) but not L status (p = 0.294).

### Effect of adjuvant radiotherapy in high-risk EC patients of less than 70 years of age

Within the cohort, 761 EC patients younger than 70 years of age were classified as high-risk. Most patients underwent surgery only (n = 359; 47.2%), whereas the remaining patients received either surgery with additional radiotherapy (n = 219; 28.8%) or a combination of surgery, radiotherapy and chemotherapy (n = 183; 24.0%).

Table 3 presents the corresponding patient characteristics.

**Table 3:** Characteristics of EC patients at high-risk

	Total	Treatment		
		SX (%)	SX + RT (%)	SX + RCT (%)
Age				
< 70 years	761	359 (47)	219 (29)	183 (24)
70 + years	1018	621 (61)	323 (32)	74 (7)
N status				
N0	935	527 (56)	296 (32)	112 (12)
N1	714	353 (49)	221 (31)	140 (20)
Not known	130	100 (77)	25 (19)	5 (4)
V status				
V0	1275	684 (54)	394 (31)	197 (15)
V1	333	186 (56)	96 (29)	51 (15)
Not known	171	110 (64)	52 (30)	9 (5)
L status				
L0	793	451 (57)	231 (29)	111 (14)
L1	824	425 (52)	262 (32)	137 (17)
Not known	162	104 (64)	49 (30)	9 (6)
Grading				
G1	183	101 (55)	58 (32)	24 (13)
G2	467	235 (50)	170 (36)	62 (13)
G3	993	559 (56)	276 (28)	158 (16)
Not known	136	85 (63)	38 (28)	13 (10)
Histology				
Endometrioid	844	423 (50)	277 (33)	144 (17)
Non-endometioid	935	557 (60)	265 (28)	113 (12)
Total	1779	980 (55)	542 (30)	257 (14)

*SX* surgery, *RCT* radio-chemotherapy, *RT* radiotherapy, *N status* nodal involvement status, *V status* vascular status, *L status* lymphovascular status

**Fig. 4:**
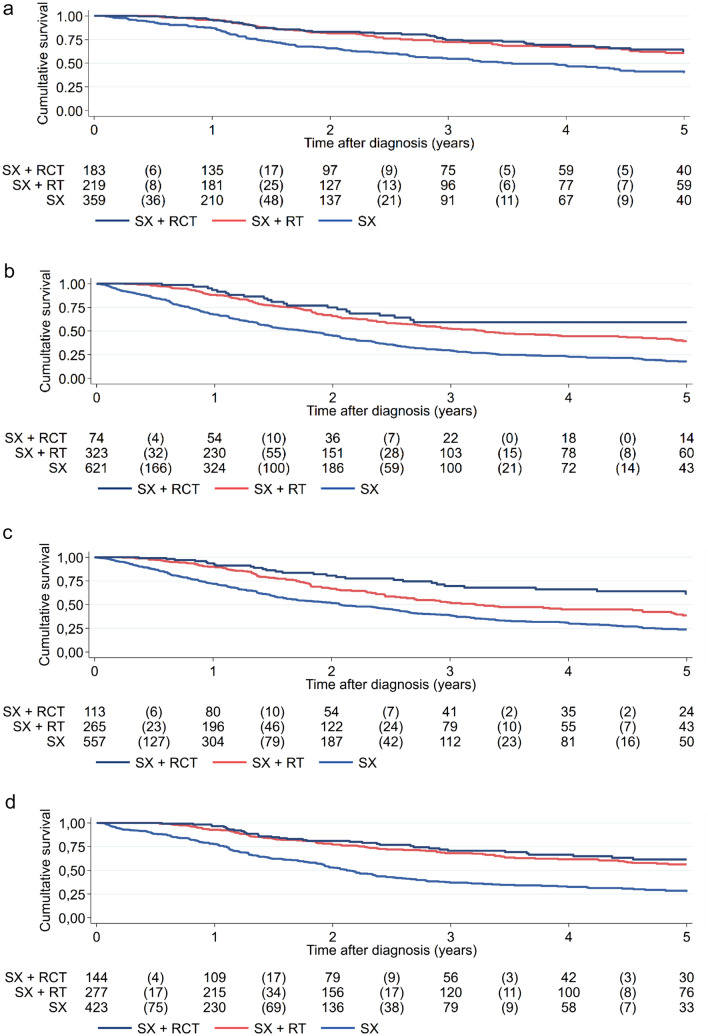

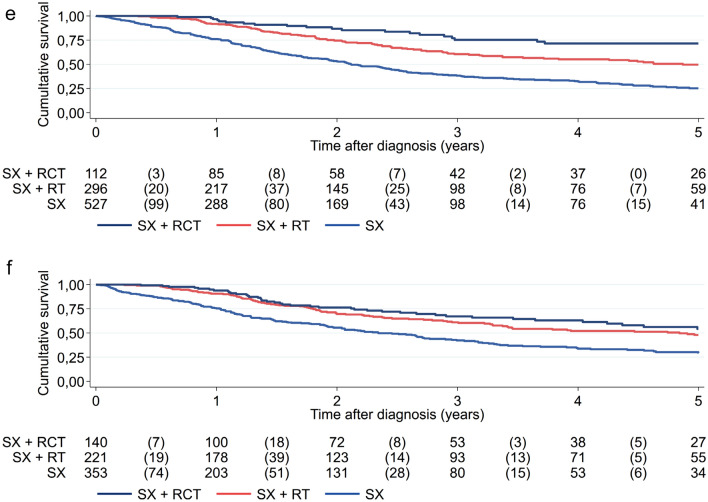
Overall survival of high-risk EC patients of less than 70 years of age (**a**), over 70 years of Age (**b**), with non-endometrioid subtype (**c**), endometrioid subtype (**d**), without nodal involvement (**e**) and with nodal involvement (**f**), who underwent surgery with or without adjuvant therapies. *SX* surgery, *RT* radiotherapy, *RCT* radiochemotherapy

Univariable survival analysis showed that a combination of therapeutic interventions increases the overall survival (s. Fig. 4a). In comparison to the combination of surgery with radiotherapy, surgery alone led to a poorer overall survival (HR = 1.903; 95% CI = 1.417–2.557; p ≤ 0.001), whereas the further addition of chemotherapy did not result in any benefit (HR = 0.914; 95% CI = 0.627–1.327; p = 0.635). Throughout the analysis period, overall survival was improved in patients receiving multimodal therapy compared to patients only receiving surgery at any measuring point (s. Supplementary Table 3).

This was in agreement with the multivariable statistical analysis (s. Supplementary Table 4). Compared to the combination of surgery with radiotherapy, surgery-only led to a poorer overall survival (Cox; HR = 2.006; 95% CI = 1.480–2.719; p ≤ 0.001). Addition of chemotherapy showed no significant additive effect over the combination of surgery and radiotherapy (HR = 0.891; 95% CI = 0.612–1.296; p = 0.544). Furthermore, multivariable statistical analysis produced statistical significance for L status (p = 0.010) and grading (G2, p = 0.007; G3, p = 0.002), and a significant result for the N status (p = 0.026). In contrast, the analysis did not reveal any significance for V status and histological subtype.

### Effects of adjuvant therapy on overall survival for patients of at least 70 years of age with high-risk EC

Among the subpopulation of high-risk EC patients, 1,018 patients were at least 70 years of age. The majority of patients underwent surgery only (n = 621; 61.0%), and a considerable fraction was subjected to surgery with radiotherapy (n = 323; 31.7%), whereas only a small fraction received surgery and radio-chemotherapy (n = 74; 7.3%). Additional patient characteristics, such as N-, V-, L- status, grading and histology, are summarized in Table 3 according to treatment.

According to univariable survival analysis, overall survival was increased by treatment with a combination of surgery and radiotherapy compared to surgery only (HR for surgery vs. surgery and radiotherapy = 1.942; 95% CI = 1.610–2.341; p = 0.000) (s. Fig. 4b) Still, the number of cases was small (n = 74). Moreover, additional treatment with chemotherapy did further improve overall survival (HR = 0.628; 95% CI = 0.406–0.973; p = 0.037). Analysis of overall survival showed that multimodal therapy, particularly surgery and radio-chemotherapy, produced better outcomes over time (s. Supplementary Table 5).

We employed multivariable statistical analysis for further validation (s. Supplementary Table 6). The outcome of the analysis showed that surgery alone reduces overall survival significantly (Cox; HR = 1.889; 95% CI = 1.561–2.287; p ≤ 0.001) compared to a combination with radiotherapy. The addition of chemotherapy further improves the overall survival (HR = 0.591; 95% CI = 0.380–0.918; p = 0.019). Another finding of the multivariable statistical analysis was that positive V status (p ≤ 0.001), L status (p = 0.007), and cancer grading G3 (p = 0.001) were significant risk factors for overall survival. The analysis also showed a weak significant effect for the N status (p = 0.043). Other cancer gradings (i.e., G2) and histology did not affect the outcome.

### Effects of adjuvant therapy on overall survival for patients with high-risk EC in correlation to histological subtype and nodal status

Among the patients with high-risk EC subgroup analyses were performed for histology subtypes (n = 1779 patients) and nodal status (n = 2502 patients). 53% of patients (n = 935) in the high-risk group had a non-endometrioid EC with around two third (n = 384) were over seventy years old, had a higher grade 3 (75% vs. 35%), but less often a nodal involvement (26% vs. 56%) compared to patients with an endometrioid histology (n = 844) with 49% with an age over seventy years. In the multivariate analysis after adjusting for age, V status, L status, grading and nodal status, the addition of chemotherapy to radiotherapy after primary tumor resection in the non-endometrioid subgroup showed a significant effect on the 5-year OS with 61.5% compared to 38.6% if only radiotherapy as an adjuvant treatment modality was applied (HR 0.553; 95% CI = 0.366–0.837; p = 0.005) (s. Fig. 4c, Supplementary Table 7a). This effect was not seen in the endometrioid subgroup (HR 0.939; 95% CI = 0.648–1.361; p = 0.741) with 5-year OS of 61.5% vs 56.3% (s. Fig. 4d, Supplementary Table 7b). Adjuvant radiotherapy were in both subgroups favorable compared to surgery alone with 5-year OS for the surgery arm of 27.7% for the endometrioid subgroup (HR 2.298, 95% CI = 1.812–2.915; p < 0.001) and 23.8% for the non-endometrioid cohort (HR 1.656, 95% CI = 1.339–2.048; p < 0.001). Nodal involvement (N1) was present in 49% (n = 1220) of patients in the high-risk group. An adjuvant therapy with radiotherapy or combined radiochemotherapy was applied in 32% (n = 296) and 12% (n = 112) in patients without nodal involvement and in 31% (n = 221) and 20% (n = 140), if nodal involvement was present. In the multivariate analysis after adjusting for age, V and L status, grading and histology the addition of chemotherapy to radiotherapy in the adjuvant setting showed a benefit in patients without nodal involvement (HR 0.486; 95% CI = 0.308–0.768; p = 0.002; Supplementary Table 8a), but not in patients with nodal involvement (HR 0.781; 95% CI = 0.552–1.106; p = 0.164; Supplementary Table 8b). Adjuvant radiotherapy showed a significant better OS compared to surgery alone without nodal involvement (HR 0.485; 95% CI = 0.387–0.607; p < 0.001) and with nodal involvement (HR 0.586; 95% CI = 0.460–0.748; p < 0.001) with 5-year OS rates of 49.6% (N0) and 47.9% (N1) compared to 25.2% and 29.3% (s. Fig. 4e, f).

## Discussion

In this study, we analyzed real-world data obtained from large cohort of 34,349 EC patients documented in German federal clinical cancer registries. We described the development of surgical approaches for EC treatment during a period of 20 years and observed a clear shift from open to minimal invasive surgery after 2006 (s. Fig. 2). Similar observations were made in other countries (e.g. Great Britain: 58%) (Moss et al. 2021). Several reasons may have led to this development. Technological advancement, education and training enabled more and more surgeons to offer and perform minimal invasive surgery. Continuous improvement of care led to diagnosis of EC in earlier stages, increasing the relative proportion of these cases within the cohort, (s. Table 1). It is likely that minimal invasive surgery was performed more often in early-stage EC patients.

A number of studies reported that outcomes after minimal invasive surgery are at least as good as after open surgery for the treatment of EC (Ghezzi et al. 2010; Odetto et al. 2023; Papathemelis et al. 2020; Shanmugam et al. 2020; Togami et al. 2020), or even better (Pedra Nobre et al. 2020). This is in line with the findings of our study. Nonetheless, there are limitations to this interpretation. Patients with a higher risk EC are more likely to receive open surgery, whereas patients with early-stage EC are more likely to receive minimal invasive surgery. This could point towards a confounding effect, which we addressed by controlling for disease severity via the different risk groups in multivariable Cox regression analyses. In doing so, we observed a statistically significant difference between the ‘low risk’ group and the other groups. Regardless of the risk group, endoscopic surgery was superior to open surgery. Furthermore, our analysis showed that vascular invasion status, lymphovascular status, grading and histology are risk factors for patients who underwent surgery, pointing towards risk of disease severity as a confounder for choosing minimal invasive vs. open surgery. The observation that age is a significant and substantial risk factor, suggests that overall health and stamina of the patient are important aspects. Since minimal invasive surgery compared to open surgery may have a positive effect on overall survival due to improvement of short-term survival and perioperative mortality. Additional studies are needed to elucidate the contradictory interpretation.

The patients in this study were classified into five risk groups based on several criteria. The ‘low risk’ group contained most patients, whereas the ‘advanced metastatic” group contained the lowest number of patients, reflecting the distribution of EC in the overall population (s. Table 1). The lower the risk group, the proportion of patients who only received surgery was higher. As expected, the proportion of patients who received additional chemotherapy or radiotherapy or a combination of both was higher in the higher risk group.

‘Intermediate risk’ patients showed good outcome with a 5-year survival rate of more than 75%. This meets the reported survival rates for patients with early-stage EC (Åkesson et al. 2023; Chen et al. 2012; Gottwald et al. 2010). Since age of the patients is a well-known risk factor, often associated with comorbidities, (Scharl et al. 2022), we distinguished in the group of ‘high-risk’ patients between those younger than 70 years of age, and those who were at least 70 years of age, essentially following observations from the PORTEC-2 trial (Wortman et al. 2018). Among the younger patient group, the overall survival in ‘high-risk’ patients was lower than the one of the ‘low risk’ and ‘intermediate risk’ groups; however, there was a clear difference depending on treatment. Patients who underwent surgery only showed the poorest outcome with a 5-year survival rate of about 40%. In contrast, the addition of radiotherapy improved the outcome significantly and the 5-year survival rate rose to about 60%. Notably, the further addition of chemotherapy did not further improve the outcome. This suggests that ‘high-risk; EC patients benefit from a combination of radiotherapy and surgery. Earlier studies support this observation (Scharl et al. 2021, 2022; Smogeli et al. 2018).

The outcome in the older ‘high-risk’ patient group was poorer in general; nonetheless, the impact of the different adjuvant therapies was pronounced. Surgery only was associated with a 5-year survival of about 20%, whereas the addition of radiotherapy roughly doubled the overall survival rate, yielding a 5-year survival rate of about 40%. Remarkably, the further addition of chemotherapy increased the survival rate even further to almost 60%. Previous studies provided some early evidence of this observation (Boer et al. 2018; Scharl et al. 2021; Smogeli et al. 2018). This suggests, that a subgroup of elderly patients with ‘high-risk’ EC may benefit from a multimodal therapy. Still, this study group was very small compared to the surgery-only group (n = 74 vs. n = 374, respectively). A weakness of the study was that patient derived data could not be adjusted in terms of comorbidities or performance status. Patients in the adjuvant radiochemotherapy arm were probably fitter than those in the surgery-only or adjuvant radiotherapy arm (Scharl et al. 2022). Thus, predictive variables may be prevalent morbidity and the performance status (Scharl et al. 2022) or histomorphological and molecular features (Boer et al. 2018).

### Strengths and limitations of the study

This is the largest study on clinical care of EC in Germany. Compared to previous studies, our analysis included several patient subgroups. Scharl et al. did not perform a selective examination of young versus old or radiochemotherapy treated versus chemotherapy treated patients (Scharl et al. 2021, 2022). Smogeli et al. only focussed on chemotherapy and did not compare with radiochemotherapy or radiotherapy (Smogeli et al. 2018).

We acknowledge that our study has certain limitations. Patients were grouped into five different risk groups based on several criteria, including cancer stage, grading, histology as well as LVSI, myometrial invasion and R status. Data on the current state-of-the-art molecular classification were not available. Furthermore, informations on recurrences, especially on specific sites were not completely available. Therefore recurrence free survival that may be a preferred outcome measure for certain questions was not applicable.

## Conclusion

Almost two thirds of EC cases have an excellent prognosis upon surgery and localized radiotherapy. Minimal invasive surgery seems safe for low risk (and intermediate) EC. The approach may even improve OS. More data is needed for a final conclusion. For treatment of high-risk EC patients, physicians overall choose a more aggressive intervention and employ an increased number of systemic therapies. The presented data indicates that multimodal therapy seems to improve the outcome, even for elderly high-risk EC patients. Our cohort analysis demonstrates the importance of evaluating clinical studies in their implementation in daily routine. Patients enrolled in clinical trials are usually highly selected for fitness and absent of severe secondary diseases. Especially in an elderly patient cohort like EC, the efficacy of treatment results may differ in the daily routine patient cohort. For high-risk patients the 5-year OS was 39 to 62% in our current analysis, while in the PORTEC-3 study distinct higher 5-yer OS of 77 to 82% was observed (Boer et al. 2018).

Additional studies including molecular classifications are needed to identify patients that are at high-risk for recurrence and may benefit from a multimodal therapy.

### Supplementary Information

Below is the link to the electronic supplementary material.

Supplementary file1 (DOCX 107 kb)

## Data Availability

The datasets generated during and/or analyzed during the current study are available from the corresponding author on reasonable request.
