# Supplementary material for: Treatment of endometrial cancer from 2000 to 2020 in Germany: a retrospective population based cohort study
Source: J Cancer Res Clin Oncol. 2024 May 27;150(5):279. doi: 10.1007/s00432-024-05772-9 (PMC11129991; doi:10.1007/s00432-024-05772-9)
Supplement: Supplementary file 1 — Supplementary file1 (DOCX 107 kb) [file 432_2024_5772_MOESM1_ESM.docx]

**SUPPLEMENTARY INFORMATION**
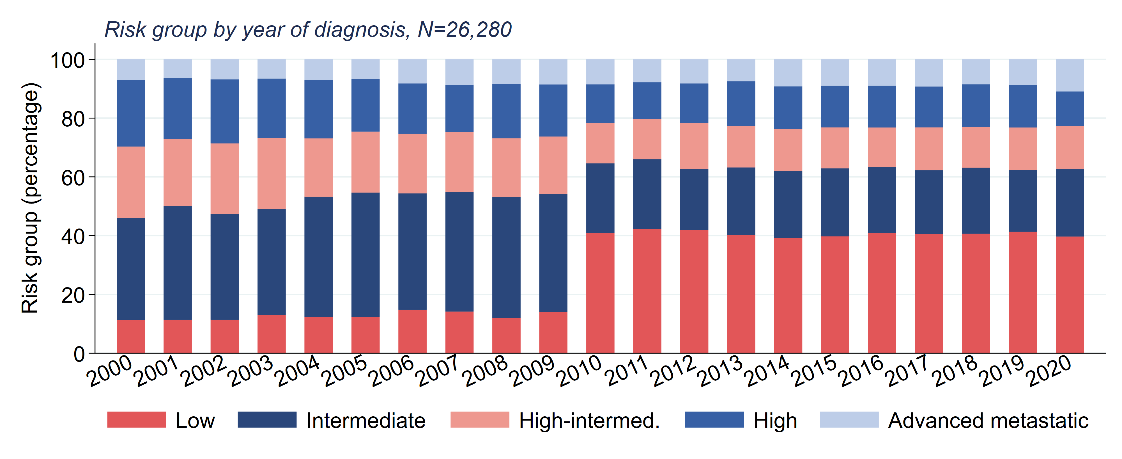


**Supplementary Figure 1**. Risk group distribution of 26,280 EC patients from 2000 to 2020.

**Supplementary Table 1. Risk group stratification**

| Risk group | Stage | Grading | Histology | LVSI | Myometrial invasion | Residual disease |
| --- | --- | --- | --- | --- | --- | --- |
| **Low** | | |  |  |  |  |
| 1 | IA | G1-G2 | endometrioid | - |  |  |
| **Intermediate** | | |  |  |  |  |
| 2 | IB | G1-G2 | endometrioid | - |  |  |
| 2 | IA | G3 | endometrioid | - |  |  |
| 2 | IA |  | serous, clear cell, un-differentiated carcinoma, carcinosarcoma, mixed |  |  |  |
| **High-intermediate** | | |  |  |  |  |
| 3 | I |  | endometrioid | + |  |  |
| 3 | IB | G3 | endometrioid |  |  |  |
| 3 | II |  |  |  |  |  |
| **High** | | |  |  |  |  |
| 4 | I-IVA |  | serous, clear cell, un-differentiated carcinoma, carcinosarcoma, mixed |  | + | R0 |
| 4 | III/IVA |  |  |  |  | R0 |
| **Advanced metastatic** | | |  |  |  |  |
| 5 | III/IVA |  |  |  |  | R1 |
| 5 | IVB |  |  |  |  |  |

LVSI, lymphovascular space invasion

**Supplementary Table 2. Overall survival in cumulative parts per year for patients subjected to open or endoscopic surgery**

| Surgery | Overall survival [year] | | | | | |
| --- | --- | --- | --- | --- | --- | --- |
|  | 1 | 2 | 3 | 4 | 5 | 95 % CI 5 years |
| Open | 0.871 | 0.736 | 0.636 | 0.571 | 0.523 | 0.496 – 0.550 |
| Endoscopic | 0.958 | 0.894 | 0.822 | 0.767 | 0.725 | 0.693 – 0.755 |

**Supplementary Table 3. Overall survival in cumulative parts per year for patients of less than 70 years of age subjected to multimodal therapy**

| Therapy | Overall survival [year] | | | | | |
| --- | --- | --- | --- | --- | --- | --- |
|  | 1 | 2 | 3 | 4 | 5 | 95 % CI 5 years |
| SX + RT | 0.959 | 0.815 | 0.723 | 0.674 | 0.609 | 0.522 – 0.685 |
| SX | 0.870 | 0.658 | 0.546 | 0.475 | 0.328 | 0.475 – 0.402 |
| SX + RCT | 0.960 | 0.833 | 0.748 | 0.695 | 0.629 | 0.526 – 0.715 |

SX, surgery; RCT, radio-chemotherapy; RT, radiotherapy

**Supplementary Table 4. Effect of therapy and risk factors on overall survival in high-risk, less than 70 years of age EC patients**

|  | HR | 95% CI | | p-value |
| --- | --- | --- | --- | --- |
| Therapy |  |  |  |  |
| SX + RT | 1.000 |  |  |  |
| SX | 2.006 | 1.480 | 2.719 | <0.001 |
| SX + RCT | 0.891 | 0.612 | 1.296 | 0.544 |
| N status |  |  |  |  |
| N0 | 1.000 |  |  |  |
| N1 | 1.070 | 0.8000618 | 1.430 | 0.649 |
| not known | 1.874 | 1.07992 | 3.252 | 0.026 |
| V status |  |  |  |  |
| V0 | 1.000 |  |  |  |
| V1 | 1.192 | 0.8581914 | 1.655 | 0.295 |
| not known | 1.509 | 0.6405438 | 3.556 | 0.347 |
| L status |  |  |  |  |
| L0 | 1.000 |  |  |  |
| L1 | 1.500 | 1.101711 | 2.043 | 0.010 |
| not known | 0.979 | 0.3792848 | 2.526 | 0.965 |
| Grading |  |  |  |  |
| G1 | 1.000 |  |  |  |
| G2 | 2.140 | 1.225216 | 3.737 | 0.007 |
| G3 | 2.410 | 1.398947 | 4.151 | 0.002 |
| not known | 2.201 | 1.074026 | 4.511 | 0.031 |
| Histology |  |  |  |  |
| endometrioid | 1.000 |  |  |  |
| non-endometrioid | 0.983 | 0.7263713 | 1.330 | 0.910 |

SX, surgery; RCT, radio-chemotherapy; RT, radiotherapy; N status, nodal status, L status, lymphovascular status; V status, vascular status

**Supplementary Table 5. Overall survival in cumulative parts per year for patients of at least 70 years of age subjected to multimodal therapy**

| Therapy | Overall survival [year] | | | | | |
| --- | --- | --- | --- | --- | --- | --- |
|  | 1 | 2 | 3 | 4 | 5 | 95 % CI 5 years |
| SX + RT | 0.882 | 0.656 | 0.525 | 0.593 | 0.393 | 0.326 – 0.460 |
| SX | 0.676 | 0.451 | 0.295 | 0.295 | 0.180 | 0.141 – 0.222 |
| SX + RCT | 0.933 | 0.749 | 0.593 | 0.593 | 0.593 | 0.443 – 0.715 |

SX, surgery; RCT, radio-chemotherapy; RT, radiotherapy

**Supplementary Table 6 Effect of therapy and risk factors on overall survival in high-risk, more than 70 years of age EC patients**

|  | HR | 95% CI | | p-value |
| --- | --- | --- | --- | --- |
| Therapy |  |  |  |  |
| SX + RT | 1.000 |  |  |  |
| SX | 1.889 | 1.561 | 2.287 | <0.001 |
| SX + RCT | 0.591 | 0.380 | 0.918 | 0.019 |
| N status |  |  |  |  |
| N0 | 1.000 |  |  |  |
| N1 | 1.021 | 0.838 | 1.243 | 0.839 |
| not known | 1.330 | 1.009 | 1.752 | 0.043 |
| V status |  |  |  |  |
| V0 | 1.000 |  |  |  |
| V1 | 1.496 | 1.203 | 1.859 | <0.001 |
| not known | 0.789 | 0.477 | 1.305 | 0.356 |
| L status |  |  |  |  |
| L0 | 1.000 |  |  |  |
| L1 | 1.315 | 1.077 | 1.606 | 0.007 |
| not known | 2.047 | 1.211 | 3.461 | 0.008 |
| Grading |  |  |  |  |
| G1 | 1.000 |  |  |  |
| G2 | 1.216 | 0.828 | 1.786 | 0.320 |
| G3 | 1.835 | 1.263 | 2.666 | 0.001 |
| not known | 1.566 | 0.985 | 2.488 | 0.058 |
| Histology |  |  |  |  |
| endometrioid | 1.000 |  |  |  |
| non-endometrioid | 0.934 | 0.765 | 1.140 | 0.505 |

SX, surgery; RCT, radio-chemotherapy; RT, radiotherapy; N status, nodal status, L status, lymphovascular status; V status, vascular status

**Supplementary Table 7a Effect of therapy and risk factors on overall survival in high-risk EC patients with non-endometrioid histology**

|  | HR | 95% CI | | p-value |
| --- | --- | --- | --- | --- |
| Therapy |  |  |  |  |
| SX + RT | 1.000 |  |  |  |
| SX | 1.613 | 1.301 | 2.000 | <0.001 |
| SX + RCT | 0.553 | 0.366 | 0.837 | 0.005 |
| Age |  |  |  |  |
| <70 years | 1.000 |  |  |  |
| 70+ years | 1.983 | 1.592 | 2.470 | <0.001 |
| N status |  |  |  |  |
| N0 | 1.000 |  |  |  |
| N1 | 1.051 | 0.835 | 1.321 | 0.673 |
| not known | 1.305 | 0.954 | 1.784 | 0.095 |
| V status |  |  |  |  |
| V0 | 1.000 |  |  |  |
| V1 | 1.772 | 1.381 | 2.275 | <0.001 |
| not known | 0.619 | 0.263 | 1.461 | 0.274 |
| L status |  |  |  |  |
| L0 | 1.000 |  |  |  |
| L1 | 1.119 | 0.892 | 1.402 | 0.331 |
| not known | 2.602 | 0.919 | 5.308 | 0.076 |
| Grading |  |  |  |  |
| G1 | 1.000 |  |  |  |
| G2 | 1.367 | 0.631 | 2.962 | 0.428 |
| G3 | 2.405 | 1.182 | 4.893 | 0.015 |
| not known | 2.074 | 0.981 | 4.385 | 0.056 |
|  |  |  |  |  |

SX, surgery; RCT, radio-chemotherapy; RT, radiotherapy; N status, nodal status, L status, lymphovascular status; V status, vascular status

**Supplementary Table 7b Effect of therapy and risk factors on overall survival in high-risk EC patients with endometrioid histology**

|  | HR | 95% CI | | p-value |
| --- | --- | --- | --- | --- |
| Therapy |  |  |  |  |
| SX + RT | 1.000 |  |  |  |
| SX | 2.410 | 1.891 | 3.071 | <0.001 |
| SX + RCT | 0.939 | 0.648 | 1.361 | 0.741 |
| Age |  |  |  |  |
| <70 years | 1.000 |  |  |  |
| 70+ years | 1.933 | 1.536 | 2.432 | <0.001 |
| N status |  |  |  |  |
| N0 | 1.000 |  |  |  |
| N1 | 1.035 | 0.815 | 1.314 | 0.777 |
| not known | 1.898 | 1.263 | 2.852 | 0.002 |
| V status |  |  |  |  |
| V0 | 1.000 |  |  |  |
| V1 | 1.096 | 0.837 | 1.435 | 0.503 |
| not known | 0.950 | 0.559 | 1.614 | 0.849 |
| L status |  |  |  |  |
| L0 | 1.000 |  |  |  |
| L1 | 4.846 | 1.422 | 2.395 | <0.001 |
| not known | 2.183 | 1.211 | 3.934 | 0.009 |
| Grading |  |  |  |  |
| G1 | 1.000 |  |  |  |
| G2 | 1.557 | 1.099 | 2.205 | 0.013 |
| G3 | 1.881 | 1.320 | 2.681 | <0.001 |
| not known | 1.550 | 0.722 | 3.328 | 0.261 |
|  |  |  |  |  |

SX, surgery; RCT, radio-chemotherapy; RT, radiotherapy; N status, nodal status, L status, lymphovascular status; V status, vascular status

**Supplementary Table 8a Effect of therapy and risk factors on overall survival in high-risk EC patients without nodal involvement**

|  | HR | 95% CI | | p-value |
| --- | --- | --- | --- | --- |
| Therapy |  |  |  |  |
| SX + RT | 1.000 |  |  |  |
| SX | 2.017 | 1.606 | 2.534 | <0.001 |
| SX + RCT | 0.587 | 0.369 | 0.935 | 0.025 |
| Age |  |  |  |  |
| <70 years | 1.000 |  |  |  |
| 70+ years | 2.024 | 1.595 | 2.568 | <0.001 |
| V status |  |  |  |  |
| V0 | 1.000 |  |  |  |
| V1 | 1.534 | 1.170 | 2.012 | 0.002 |
| not known | 1.528 | 0.738 | 3.162 | 0.254 |
| L status |  |  |  |  |
| L0 | 1.000 |  |  |  |
| L1 | 1.357 | 1.079 | 1.706 | 0.009 |
| not known | 1.044 | 0.483 | 2.258 | 0.913 |
| Grading |  |  |  |  |
| G1 | 1.000 |  |  |  |
| G2 | 1.349 | 0.822 | 2.215 | 0.237 |
| G3 | 1.724 | 1.055 | 2.819 | 0.030 |
| not known | 1.666 | 0.952 | 2.917 | 0.074 |
| Histology |  |  |  |  |
| endometrioid | 1.000 |  |  |  |
| non-endometrioid | 1.060 | 0.819 | 1.373 | 0.655 |
|  |  |  |  |  |

SX, surgery; RCT, radio-chemotherapy; RT, radiotherapy; N status, nodal status, L status, lymphovascular status; V status, vascular status

**Supplementary Table 8b Effect of therapy and risk factors on overall survival in high-risk EC patients with nodal involvement**

|  | HR | 95% CI | | p-value |
| --- | --- | --- | --- | --- |
| Therapy |  |  |  |  |
| SX + RT | 1.000 |  |  |  |
| SX | 1.732 | 1.353 | 2.217 | <0.001 |
| SX + RCT | 0.897 | 0.627 | 1.283 | 0.552 |
| Age |  |  |  |  |
| <70 years | 1.000 |  |  |  |
| 70+ years | 1.920 | 1.530 | 2.409 | <0.001 |
| V status |  |  |  |  |
| V0 | 1.000 |  |  |  |
| V1 | 1.224 | 0.935 | 1.604 | 0.142 |
| not known | 0.699 | 0.375 | 1.303 | 0.259 |
| L status |  |  |  |  |
| L0 | 1.000 |  |  |  |
| L1 | 1.542 | 1.159 | 2.051 | 0.003 |
| not known | 2.602 | 1.296 | 5.221 | 0.007 |
| Grading |  |  |  |  |
| G1 | 1.000 |  |  |  |
| G2 | 1.629 | 1.014 | 2.618 | 0.044 |
| G3 | 2.381 | 1.497 | 3.785 | <0.001 |
| not known | 1.355 | 0.634 | 2.895 | 0.433 |
| Histology |  |  |  |  |
| endometrioid | 1.000 |  |  |  |
| non-endometrioid | 0.913 | 0.717 | 1.162 | 0.459 |
|  |  |  |  |  |

SX, surgery; RCT, radio-chemotherapy; RT, radiotherapy; N status, nodal status, L status, lymphovascular status; V status, vascular status
